# Editorial: Advances in multi-omics study of filamentous plant pathogens

**DOI:** 10.3389/fmicb.2022.998501

**Published:** 2022-08-12

**Authors:** Danyu Shen, Yan Wang, Xiaoren Chen, Vaibhav Srivastava, Silvia Laura Toffolatti

**Affiliations:** ^1^Department of Plant Pathology, Nanjing Agricultural University, Nanjing, China; ^2^College of Horticulture and Plant Protection, Yangzhou University, Yangzhou, China; ^3^Division of Glycoscience, Department of Chemistry, School of Engineering Sciences in Chemistry, Biotechnology and Health, KTH Royal Institute of Technology, AlbaNova University Center, Stockholm, Sweden; ^4^Dipartimento di Scienze Agrarie ed Ambientali, Università degli Studi di Milano, Milan, Italy

**Keywords:** omics, oomycetes, fungi, pathogenesis, plant-pathogen interaction

Filamentous plant pathogens, such as fungi and oomycetes, cause devastating diseases of crop plants, and result in serious threats to agriculture and natural ecosystems worldwide. Diseases caused by fungal pathogens, such as wheat stem rust (*Puccinia graminis*), rice blast (*Magnaporthe oryzae*), and gray mold (*Botrytis cinerea*), are of immediate concern for global food security. The oomycete *Phytophthora infestans*, the causal agent of the Great Irish Famine in the nineteenth century, remains a recurring threat to potato and tomato production today. *P. sojae* causes soybean root and stem rot, while *P. capsici* infects a large number of agriculturally important vegetables, resulting in severe yield losses. In order to control such plant diseases, the broad spectrum of the infection process and pathogenic mechanisms that filamentous pathogens apply to colonize plants needs to be elucidated. However, there are still many gaps in our current understanding. High-throughput technologies have revolutionized plant pathology research. Omics technologies, such as genomics, transcriptomics, proteomics, metabolomics, epigenomics, and microbiomics, have become essential resources in the plant-pathogen interaction research. Pathogen genomics focuses on the structure, function, and evolution of genomes, while transcriptomics and proteomics provide insights into gene and protein expression. This Research Topic aimed to collect studies of omics data to provide an increased understanding of various aspects related to plant-pathogen biology and plant-pathogen interactions. It comprises nine interesting papers covering a broad range of themes. Here, we grouped these articles into the following five themes.

## Genomics

Whole-genome sequencing is a comprehensive approach for analyzing entire genomes, and thus is significantly helpful for biological research. Mandal et al. analyzed 128 *Phytophthora* genomes, and found that the simple sequence repeats of all studied *Phytophthora* species followed distinct isolate-specific patterns. The pathogenesis-related genes were localized in more repeat-rich regions, and RxLR effectors diverged rapidly and did not show any common core group. Nellist et al. performed comparative genomic analysis of 18 *Phytophthora cactorum* isolates, and these isolates showed specialization in strawberry crown and apple. This study also highlighted several RxLR effectors that warrant further investigation as host specificity determinants. Wyrebek et al. evaluated the value of mitogenome to identify *Fusarium graminearum sensu stricto* (*F.g*.). They found that homing endonucleases displayed small indel polymorphism, which facilitated further distinguishing between *F.g*. and related species belonging to *F. graminearum* species complex.

## Transcriptomics

Transcriptome sequencing provides data on the expression of almost all transcripts in a certain state, and has been widely used in the study of plant-pathogen interactions. Daly et al. performed a dual-transcriptomic analysis of the interaction between the biocontrol agent *Pythium oligandrum* and ginger pathogen *Pythium myriotylum*, and found that *Py. myriotylum* genes encoding Kazal-type protease inhibitors, cellulases, and elicitin-like proteins were strongly up-regulated, while *Py. oligandrum* genes coding for proteases, cellulases, and peroxidases were highly activated. Tang et al. investigated transcriptome variations of *Verticillium dahliae* in response to two different inorganic nitrogen sources. They found that genes related to oxidoreductase, cell cycle, and some metabolic activities were down-regulated in the ammonium treatment. Meanwhile, differentially expressed genes related to glycerol transport, energy-dependent multidrug efflux pump activity, and L-serine biosynthesis, were involved in the utilization of both nitrate and ammonium.

## Proteomics

Post-translational modifications, such as redox modification, are involved in many biological processes and physiological pathways in both eukaryotes and prokaryotes. However, protein redox modification in phytopathogenic fungi is unclear. Zhang et al. surveyed thiol proteome using a mixed sample containing mycelia with or without oxidative stress, invasive hyphae, conidia, and appressoria of the model fungal pathogen *Magnaporthe oryzae*, and identified the thiol-modified proteins by protein domain, functional classification, subcellular localization, metabolic pathways, and protein–protein interaction network analyses. Their findings indicated that redox modification may play key roles in fungal growth, conidium formation, appressorium formation, and invasive growth. This study provides a global insight into the redox proteome of the plant pathogenic fungi and is a valuable resource for future studies on redox modification in fungi.

## Metabolomics

Metabolism is the set of biochemical reactions that occur in living organisms. During plant-microbe interactions, extensive changes occur in plant and microbe metabolism involved in many molecular and cellular processes taking place within cells. Genome-scale metabolic models (GEMs) have become an important tool for systematically analyzing metabolic alterations associated with plant-microbe interactions. Due to their integrative nature, by relating genes with reactions and metabolites, GEMs can simulate the system-wide metabolic fluxes of both the plant and pathogen, and help identify genes, reactions, and metabolites essential for understanding the mechanisms underlying plant-microbe interactions. Rodenburg et al. reviewed the current knowledge applying metabolic modeling to dissect microbial pathogenesis and host–pathogen interactions and proposed a workflow for reconstructing high-quality GEMs.

## Functional studies

The mevalonate (MVA) pathway is involved in the biosynthesis of isoprenoids and sterols, but the information is limited in the sterol-auxotrophic *Phytophthora* species. Yang et al. firstly identified the whole set of MVA pathway genes in *Phytophthora sojae in silico*. Then, they explored the functions of MVA pathway by treatment with enzyme inhibitor lovastatin, deletion of the geranylgeranyl diphosphate synthase gene *PsBTS1*, and transcriptome analysis. They discovered that the MVA pathway played essential roles in vegetative growth, development, sexual and asexual reproduction and virulence in *P. sojae*. He et al. characterized the functions of the transcription factor *VpxlnR* in *Valsa pyri*. Through creating gene deletion mutants, they discovered that *VpxlnR* was involved in fruiting body differentiation, growth on culture media, response to chemical stress (hydrogen peroxide and salicylic acid) and infection of detached pear leaves and branches at the early infection stages. Furthermore, they identified five target genes that could interact with VpxlnR *in vivo* based on a yeast one-hybrid assay, providing useful information about the mechanism of pathogenesis of *V. pyri*.

## Author contributions

DS, YW, XC, VS, and ST co-edited the Research Topic and wrote, edited, and approved the final version of the editorial.

## Funding

DS was funded by the National Natural Science Foundation of China (NSFC; 32070139), and Jiangsu Agricultural Science and Technology Innovation Fund [CX(21)3085]. XC was funded by NSFC (31871907, 31671971) and Jiangsu Agriculture Science and Technology Innovation Fund (JASTIF) (CX(20)3125). VS was funded by European Commission (Horizon 2020 FET-OPEN Grant Agreement No. 828940) and the Swedish Research Council FORMAS (Grant #2019-00912).

## Conflict of interest

The authors declare that the research was conducted in the absence of any commercial or financial relationships that could be construed as a potential conflict of interest.

## Publisher's note

All claims expressed in this article are solely those of the authors and do not necessarily represent those of their affiliated organizations, or those of the publisher, the editors and the reviewers. Any product that may be evaluated in this article, or claim that may be made by its manufacturer, is not guaranteed or endorsed by the publisher.

